# Incidence of Coronary Artery Disease in King Abdulaziz University Hospital, Jeddah, Saudi Arabia, 2019–2020: A Retrospective Cohort Study

**DOI:** 10.7759/cureus.28770

**Published:** 2022-09-04

**Authors:** Fatma Albeladi, Iman Wahby Salem, Mohammed Zahrani, Layal Alarbedi, Abdulrahman Abukhudair, Huda Alnafei, Abeer Alraiqi, Nourah Alyoubi

**Affiliations:** 1 Nephrology, Faculty of Medicine, King Abdulaziz University, Jeddah, SAU; 2 Community Medicine, King Abdulaziz University, Jeddah, SAU; 3 Internal Medicine, King Abdulaziz University, Jeddah, SAU; 4 Medicine and Surgery, Faculty of Medicine, King Abdulaziz University, Rabigh, SAU

**Keywords:** saudi arabia, cardiovascular disease, coronary artery disease, epidemiology, incidence

## Abstract

Background

Coronary artery disease (CAD) remains a significant cause of death and morbidity in people globally despite advances in treatment. Prevention of CAD risk factors is crucial to reducing its prevalence. We conducted this study to determine the incidence of CAD from 2019 to 2020 in King Abdulaziz University Hospital (KAUH), Jeddah, Kingdom of Saudi Arabia (KSA), and its major risk factors among this population.

Method

This retrospective study involved all patients diagnosed with CAD at KAUH in 2019 and 2020. We analyzed validated hospital data to determine the incidence of CAD and identify the risk factors among participants. The incidence of CAD was calculated based on the total number of patients admitted to KAUH by gender, age group, and nationality (Saudi/non-Saudi).

Result

The study included 1,364 patients with a mean age of 49 years. Most patients were men (n=1,050; 77%), with fewer women (n=314; 23%), and 71.2% were non-Saudi. The incidence of CAD in 2019 was 220.98 per 10,000, and the incidence in 2020 was 3,030.52 per 10,000. However, the incidence for 2020 was confounded by the coronavirus disease 2019 pandemic-related restrictions affecting hospital admissions. The most common diagnosis was acute transmural myocardial infarction, and patients aged <60 years had a significantly high incidence of hypertension, high total cholesterol levels, low low-density lipoprotein levels, and high triglyceride levels. Patients ≥60 years had a significantly high incidence of chronic kidney disease, low hemoglobin levels, history of ischemic heart disease, and intensive care unit or critical care unit admission.

Conclusion

The study demonstrated a significant rise in CAD incidence associated with advanced age and male sex. Further prevention and control of these risk factors would be essential to decrease the incidence of CAD. A national community-based prevention effort should be implemented to avoid the expected CAD epidemic in KSA.

## Introduction

Coronary artery disease (CAD) is one of the most common cardiovascular disorders worldwide. CAD is the leading cause of death in high-income and low-income countries [1]. Despite advances in preventing and managing this disease, CAD remains a challenge. By 2030, CAD will be the most serious and widespread human health hazard [2].

A decrease in blood flow to the myocardium is the final common pathway leading to death and other complications of CAD (e.g., myocardial infarction and angina). An increased prevalence of obstruction of coronary arteries due to atherosclerosis has been attributed to an age-related rise in disease severity [3]. According to World Health Organization data published in May 2014, CAD is one of the leading causes of death in Saudi Arabia, accounting for 19,569 deaths or 24.34% of total deaths [4].

According to a nationwide survey, Saudi Arabia has a CAD prevalence of 5.5%, which is higher than that in China (2%), India (3%), and Europe (5%) but lower than that in the USA (6.7%) and Egypt (8.3%) [5-8]. The body mass index (BMI) studies demonstrated that a waist-to-height ratio cutoff value of 0.5 was effective and that rising adiposity was substantially related to the risk of CAD [9].

Diabetes and hypertension (HTN) are two prominent risk factors that can, directly and indirectly, predict the more severe adverse outcome [10]. Although no definitive association has been shown between cigarette smoking and CAD, cigarette smoking has been connected to thrombocytic and atherogenic pathways and platelet behavior [11]. We conducted this study to assess the incidence of CAD from 2019 to 2020 in King Abdulaziz University Hospital (KAUH), Jeddah, Kingdom of Saudi Arabia, and to determine the significant risk factors for CAD.

## Materials and methods

This retrospective study involved all patients diagnosed with CAD at KAUH between 2019 and 2020. We analyzed validated hospital data to determine the incidence of CAD and related risk factors. The incidence of CAD in the present study was calculated based on the total number of patients admitted to KAUH by gender, age group, and nationality (Saudi/non-Saudi) in 2019 and 2020. The total number of patients with CAD was 1,802 of 46,574 admissions (573 per 10,000). The inclusion criteria were all patients, regardless of age and sex, diagnosed with CAD, and the exclusion criteria were all patients other than complaints of CAD or patients with incomplete medical records. A checklist was prepared to include patient demographic data, smoking habits, diabetes status, HTN, history of stroke, malignancy, chronic kidney disease (CKD), family history of CAD, past history of heart-related diseases or surgery, laboratory results, and electrocardiogram (ECG) findings.

The retrospective, noninterventional study was approved by the biomedical research unit at King Abdul Aziz University (Approval No. 629-20). The Unit of Biomedical Ethics is registered at the National Committee of Biomedical and Medical Ethics (Reg No. HA-02-J-008).

Statistical analysis

Data were analyzed using IBM SPSS Statistics for Windows, Version 26.0. (IBM Corp., Armonk, NY). Qualitative data were expressed as numbers and percentages, and the chi-squared test (χ2) was used to test the relationship between variables. Quantitative data were expressed as mean and standard deviation (mean ± SD), where the independent sample t-test was used for parametric variables. A p-value of <0.05 was considered statistically significant.

## Results

This study enrolled 1,802 patients initially and then we excluded the patients with missing data, leaving 1,364 patients in the study. Our patient population consisted of 1,050 men (77%) and 314 women (23%), with a mean age of 49 years (Table 1). Men were more commonly affected than women, and most were unemployed (n=1,030; 75.5%). A total of 917 (67.2%) participants were non-Saudi, and most were married (n=1,241; 90.9%). The mean BMI of the study population was 29.20 kg/m^2^ ± 17.597 kg/m^2^. Only 29.9% of the population of CAD patients had a healthy weight. The incidence of CAD in 2019 was 220.89 per 10,000 patients; in 2020, the incidence was 3,030.52 per 10,000 patients. However, hospital admissions were restricted in 2020 due to coronavirus disease 2019 (COVID-19) pandemic precautions, which confounded our data.

**Table 1:** Sociodemographic and lifestyle data of the participants of the studied sample

Variable	N = 1364	Percent (%)
Gender		
Male	1050	77.0%
Female	314	23.0%
Marital status		
Married	1241	90.9%
Single	123	9.0%
Nationality		
Saudi	447	32.7%
Non-Saudi	917	67.2%
Occupation		
Employed	272	19.9%
Retirement	62	4.5%
unemployed	1030	75.5%
Body mass index		
Underweight	15	1.1%
Normal	408	29.9%
Overweight	526	38.6%
Obese	415	30.4%
Changing eating habits to help lower or control blood pressure		
Yes	114	8.4%
No	1250	91.6%
Exercise or physical activity		
Yes	451	33.1%
No	686	50.3%
Cannot move	226	16.6%
Age groups		
<60 years	765	56.1%
≥60 years	599	43.9%
Years of the study		
2019	783	57.41%
2020	581	42.51%

Table 2 presents the risk factor analysis. Most patients (n=878; 64.4%) were nonsmokers, 904 (66.3%) had HTN, and 836 (61.3%) had diabetes. Only 408 patients (29.9%) had a family history of CAD, stroke, and HTN. In addition, 67 patients had a history of stroke (4.9%), 36 had active malignancy (2.6%), and 87 had CKD (6.4%). More than half the population (n=814; 59.7%) had abnormal ECG findings, and 711 (52.1%) had low hemoglobin levels. Clotting factors were within the reference range in 78 patients (4%), and 714 (52.3%) had high levels of glycated hemoglobin (HbA1c), an indicator of diabetes. A total of 889 (65.2%) patients had high troponin levels, and 831 (60.9%) had cardiac enzyme levels within reference ranges. blood cholesterol levels were within the reference range in 884 (64.8 %) patients, and 696 (51.0%) had normal levels of high-density lipoprotein (HDL). Also, 356 (26.1 %) patients had high levels of low-density lipoprotein (LDL), and 904 (66.3%) had triglycerides within reference ranges.

**Table 2:** Distribution of the participants regarding CAD risk factors Abbreviations: CAD, coronary artery disease; ECG, electrocardiogram; CK, creatine kinase; HbA1c, glycated hemoglobin; HDL, high-density lipoprotein; LDL, low-density lipoprotein.

Variable	N = 1364	Percent (%)
Smoking habits		
Yes	264	19.4%
No	878	64.4%
Former (quit smoking)	222	16.3%
Hypertension		
Yes	904	66.3%
No	460	33.7%
Diabetes		
Yes	836	61.3%
No	528	38.7%
Stroke		
Yes	67	4.9%
No	1297	95.1%
Active malignancy		
Yes	36	2.6%
No	1328	97.4%
Chronic kidney disease		
Yes	87	6.4%
No	1277	93.6%
Others		
Yes	48	3.5%
No	1316	96.5%
Family history of coronary artery, stroke, or hypertension		
Yes	408	29.9%
No	949	69.6%
ECG finding		
Normal	97	7.1%
Abnormal	814	59.7%
Not found	453	33.2%
Hemoglobin		
High	30	2.2%
Normal	610	44.7%
Low	711	52.1%
Not found	13	1.0%
Clotting factors		
High	365	26.8%
Normal	781	57.3%
Low	53	3.9%
Not found	165	12.1%
HbA1c		
High	714	52.3%
Normal	330	24.2%
Low	10	0.7%
Not found	310	22.7%
Cardiac enzyme troponin		
High	889	65.2%
Normal	313	22.9%
Low	108	7.9%
Not found	54	4.0%
Cardiac enzyme CK		
High	435	31.9%
Normal	831	60.9%
Low	20	1.5%
Not found	78	5.7%
Blood cholesterol		
High	275	20.2%
Normal	884	64.8%
Low	4	0.3%
Not found	201	14.7%
HDL		
High	43	3.2%
Normal	696	51.0%
Low	303	22.2%
Not found	322	23.6%
LDL		
High	356	26.1%
Normal	713	52.3%
Low	3	0.2%
Not found	292	21.4%
Triglycerides		
High	212	15.5%
Normal	904	66.3%
Low	4	0.3%
Not found	244	17.9%

Table 3 shows that 494 (36.2%) patients had a history of past angina pectoris, 389 (28.5%) had a myocardial infarction, and 228 (16.7%) had heart failure. Only 159 patients (11.7%) had a history of ischemic heart disease (IHD), 500 (36.7%) had a history of cardiac catheterization, and 214 (15.7%) had a history of coronary artery angioplasty. In addition, 313 (22.9%) patients had a history of percutaneous coronary intervention. Most patients (n=1267; 92.9%) had inpatient episodes, of whom 916 (67.2%) had their episodes in the coronary care unit (CCU). The most common diagnoses were acute transmural myocardial infarction (n=652; 47.8%), acute subendocardial myocardial infarction (n=377; 27.6%), and unstable angina (n=220; 16.1%).

**Table 3:** Distribution of CAD patients according to episode, diagnosis, and history Abbreviations: CAD, coronary artery disease; CCU, coronary care unit; ERU, emergency room unit; MIC, medical intensive care unit; DCU, day care unit; PT, physical therapy; SIC, surgical intensive care unit; ERO, emergency room operation; IHD, ischemic heart disease; ICU, intensive care unit; PCI, percutaneous coronary intervention.

Variable	N=1364	Percent (%)
Episode location		
CCU	916	67.2%
Female section	67	4.9%
Male section	117	8.5%
ERU	34	2.5%
MIC	49	3.6%
DCU	9	0.7%
PT	6	0.4%
SIC and ERO	48	3.5%
Other	10	0.7%
Diagnosis		
Unstable angina	220	16.1%
Acute subendocardial myocardial infarction	377	27.6%
Acute transmural myocardial infarction	652	47.8%
Angina pectoris	70	5.1%
Acute ischemic heart disease	37	2.7%
Chronic ischemic heart disease	8	0.6%
Previous angina pectoris		
Yes	494	36.2%
No	870	63.8%
Previous myocardial infraction		
Yes	389	28.5%
No	975	71.5%
Previous heart failure		
Yes	228	16.7%
No	1136	83.3%
Past IHD		
Yes	159	11.7%
No	1205	88.3%
History of cardiac catheterization		
Yes	500	36.7%
No	864	63.3%
Coronary angioplasty		
Yes	214	15.7%
No	1150	84.3%
Coronary artery bypass surgery		
Yes	62	4.5%
No	1302	95.5%
PCI		
Yes	313	22.9%
No	1051	77.1%
Episode type		
Inpatient	1267	92.9%
Emergency	97	7.1%
History of ICU admission or CCU		
Yes	345	26%
No	1010	74%

In our study population, 646 (84.4%) patients younger than age 60 years were males, and 195 patients aged 60 or older (32.6%) were females (Table 4). In addition, 272 (19.9%) patients were employed (p<0.05), while a significant number of those aged 60 or older were Saudi (p<0.05). A total of 311 (40.7%) patients younger than 60 years were overweight, and significantly more patients younger than 60 were smokers than those older than age 60 (p<0.05). We found no significant difference between the two age groups regarding changes in eating habits to help lower or control blood pressure, exercise, physical activity, or diagnoses (p>0.05).

**Table 4:** Relationship between participant age groups and their characteristics and special habits Abbreviation: BMI, body mass index.

Variable	Age < 60 years (n=765)	Age ≥ 60 years (n=599)	p-Value
Gender			
Male	646 (84.4%)	404 (67.4%)	0.00
Female	119 (15.6%)	195 (32.6%)	0.00
Nationality			
Saudi	220 (28.8%)	227 (37.9%)	0.00
Non-Saudi	545 (71.2%)	372 (62.1%)	0.00
Marital status			
Married	687 (89.8%)	554 (92.5%)	0.08
Unmarried	78 (10.2%)	45 (7.5%)	0.08
Occupation			
Employed	170 (22.2%)	102 (17.0%)	0.03
Retirement	566 (74.0%)	464 (77.5%)	0.03
Uunemployed	29 (3.8%)	33 (5.5%)	0.03
BMI			
Underweight	5 (0.7%)	10 (1.7%)	0.05
Normal weight	232 (30.3%)	176 (29.4%)	0.05
Overweight	311 (40.7%)	215 (35.9%)	0.05
Obesity	217 (28.4%)	198 (33.1%)	0.05
Smoking habits			
Yes	180 (23.5%)	84 (14.0%)	0.00
No	458 (59.9%)	420 (70.1%)	0.00
Former smoker	127 (16.6 %)	95 (15.9%)	0.00
Exercise			
Yes	266 (34.8%)	185 (30.9%)	0.00
No	387 (50.6%)	299 (49.9%)	0.00
Rest	112 (14.6%)	114 (19.0%)	0.00
Changing eating habits to help lower or control blood pressure			
Yes	59 (7.7%)	55 (9.2%)	0.33
No	706 (92.3%)	544 (90.8%)	0.33

Patients aged 60 or older (i.e., older patients) had a significantly higher mean height compared to those younger than 60 years (i.e., younger patients; p<0.05; Table 5). However, younger patients had a significantly higher mean weight than older patients.

**Table 5:** Mean height and weight by age group

Variable	Age group		p-Value
	<60 years (n=765)	>60 years (n=599)	
Mean height ± SD	165.94 ± 9.93 cm	163.02 ± 9.42 cm	0.00
Mean weight ± SD	80.36 ± 49.90 kg	75.32 ± 15.41 kg	0.02

Significantly more older patients had low hemoglobin levels (n=342; 57.1%) than younger patients (p<0.05; Table 6). Significantly more younger patients had high cholesterol levels (24.2%), lower LDL levels (0.3%), and higher triglyceride levels (17.9%; p<0.05) than the older patients. More younger patients were smokers and had HTN than older patients (p≤0.05). However, CKD was significantly more common in older patients than the younger patients (p<0.05). We found no significant difference in BMI, ECG findings, clotting factors, HbA1c, HDL, cardiac enzyme troponin, and CK.

**Table 6:** Relationship between patient age and past medical/family history and laboratory findings Abbreviations: CAD, coronary artery disease; CCU, coronary care unit; ECG, electrocardiogram; HDL, high-density lipoprotein; LDL, low-density lipoprotein; ERU, emergency room unit; MIC, medical intensive care unit; DCU, day care unit; PT, physical therapy; SIC, surgical intensive care unit; ERO, emergency room operation; IHD, ischemic heart disease; ICU, intensive care unit; PCI, percutaneous coronary intervention; HbA1c, glycated hemoglobin.

Variable	Age <60 years (n=765); N (%)	Age ≥60 years (n=599); N (%)	P-Value
ECG finding			
Normal	62 (8.1%)	35 (5.8%)	0.36
Abnormal	454 (59.3%)	360 (60.1%)	0.36
Not found	249 (32.5%)	204 (34.1%)	0.36
Hemoglobin			
High	15 (2.0%)	15 (2.5%)	0.00
Normal	373 (48.8%)	237 (39.6%)	0.00
Low	369 (48.2%)	342 (57.1%)	0.00
Not found	8 (1.0%)	5 (0.8%)	0.00
Clotting factors			
High	189 (24.7%)	176 (29.4%)	0.10
Normal	444 (58.0%)	337 (56.3%)	0.10
Low	36 (4.7%)	17 (2.8%)	0.10
Not found	96 (12.5%)	69 (11.5%)	0.10
HbA1c			
High	398 (52.0%)	316 (52.8%)	0.23
Normal	199 (26.0%)	131 (21.9%)	0.23
Low	6 (0.8%)	4 (0.7%)	0.23
Not found	162 (21.2%)	148 (24.7%)	0.23
Cardiac enzyme troponin			
High	506 (66.1%)	383 (63.9%)	0.76
Normal	168 (22.0%)	145 (24.2%)	0.76
Low	63 (8.2%)	45 (7.5%)	0.76
Not found	28 (3.7%)	26 (4.3%)	0.76
Creatine kinase			
High	249 (32.5%)	186 (31.1%)	0.95
Normal	462 (60.4%)	369 (61.6%)	0.95
Low	11 (1.4%)	9 (1.5%)	0.95
Not found	43 (5.6%)	35 (5.8%)	0.95
Blood cholesterol			
High	185 (24.2%)	90 (15.0%)	0.00
Normal	476 (62.2%)	408 (68.1%)	0.00
Low	1 (0.1%)	3 (0.5%)	0.00
Not found	103 (13.5%)	98 (16.4%)	0.00
HDL			
High	26 (3.4%)	17 (2.8%)	0.46
Normal	398 (52.0%)	298 (49.7%)	0.46
Low	174 (22.7%)	129 (21.5%)	0.46
Not found	167 (21.8%)	155 (25.9%)	0.46
LDL			
High	231 (30.2%)	125 (20.9%)	0.00
Normal	384 (50.2%)	329 (54.9%)	0.00
Low	2 (0.3%)	1 (0.2%)	0.00
Not found	148 (19.3%)	144 (24.0%)	0.00
Triglycerides			
High	137 (17.9%)	75 (12.5%)	0.04
Normal	497 (65.0%)	407 (67.9%)	0.04
Low	3 (0.4%)	1 (0.2%)	0.04
Not found	128 (16.7%)	116 (19.4%)	0.04
Hypertension			
Yes	471 (61.6%)	433 (72.3%)	0.00
No	294 (38.4%)	166 (27.7%)	0.00
Diabetes			
Yes	451 (59.0%)	385 (64.3%)	0.05
No	314 (41.0%)	214 (35.7%)	0.05
Stroke			
Yes	30 (3.9%)	37 (6.2%)	0.06
No	735 (96.1%)	562 (93.8%)	0.06
Active malignancy			
Yes	21 (2.7%)	15 (2.5%)	0.88
No	744 (97.3%)	584 (97.5%)	0.88
CKD			
Yes	38 (5.0%)	49 (8.2%)	0.02
No	727 (95.0%)	550 (91.8%)	0.02
Others			
Yes	22 (2.9%)	26 (4.3%)	0.15
No	743 (97.1%)	573 (95.7%)	0.15
Family history of the coronary artery, stroke, or hypertension			
Yes	216 (28.2%)	192 (32.1%)	0.13
No	549 (71.8%)	407 (67.9%)	0.13
Previous angina pectoris			
Yes	277 (36.2%)	217 (36.2%)	0.11
No	488 (63.8%)	382 (63.8%)	0.11
Previous myocardial infraction			
Yes	209 (27.3%)	180 (30.1%)	0.37
No	556 (72.7%)	419 (69.9%)	0.37
Previous heart failure			
Yes	117 (15.3%)	111 (18.5%)	0.11
No	648 (84.7%)	488 (81.5%)	0.11
Past IHD			
Yes	77 (10.1%)	82 (13.7%)	0.04
No	688 (89.9%)	517 (86.3%)	0.04
History of cardiac catheterization			
Yes	269 (35.2%)	231 (38.6%)	0.21
No	496 (64.8%)	368 (61.4%)	0.21
Coronary angioplasty			
Yes	118 (15.4%)	96 (16.0%)	0.86
No	647 (84.6%)	503 (84.0%)	0.86
Coronary artery bypass surgery			
Yes	34 (4.4%)	28 (4.7%)	0.84
No	731 (95.6%)	571 (95.3%)	0.84
PCI			
Yes	181 (23.7%)	132 (22.0%)	0.58
No	584 (76.3%)	467 (78.0%)	0.58
Episode type			
Inpatient	63 (8.2%)	0 (0.0%)	0.00
Emergency	702 (91.8%)	599 (100.0%)	0.00
Episode location			
CCU	562 (73.5%)	354 (59.1%)	0.00
Female section	31 (4.0%)	36 (6.0%)	0.00
Male section	50 (6.5%)	67 (11.2%)	0.00
ERU	17 (2.2%)	17 (2.8%)	0.00
MIC	8 (1.0%)	41 (6.8%)	0.00
DCU	2 (0.3%)	7 (1.2%)	0.00
PT	1 (0.1%)	5 (0.8%)	0.00
SIC and ERO	23 (3.0%)	25 (4.1%)	0.00
Others hospital department	65 (8.5%)	43 (7.2%)	0.00
History of ICU admission or CCU			
Yes	174 (22.7%)	180 (30.1%)	0.00
No	591 (77.3%)	419 (69.9%)	0.00
Diagnosis			
Unstable angina	120 (15.7%)	100 (16.7%)	0.05
Acute subendocardial myocardial infarction	190 (24.8%)	187 (31.2%)	0.05
Acute transmural myocardial infarction	392 (51.2%)	260 (43.4%)	0.05
Angina pectoris	41 (5.4%)	29 (4.8%)	0.05
Acute IHD	19 (2.5%)	18 (3.0%)	0.05
Chronic IHD	3 (0.4%)	5 (0.8%)	0.05

History of IHD was significantly more common in older patients (13.7%; p≤0.05) than in younger patients. Inpatient care was significantly more common in younger patients (8.2%) than in older patients, whose care more often occurred in the CCU or ICU (p<0.05). We found no significant differences between the two age groups regarding past angina pectoris, myocardial infarction, cardiac catheterization, coronary artery bypass surgery, percutaneous coronary intervention, diabetes, stroke, malignancy, family history of CAD stroke, or HTN (p>0.05).

## Discussion

We found an incidence of CAD in 2019 of 220.89 per 10,000. The 2020 COVID-19 pandemic-related restrictions on hospital admissions confounded our data for 2020 because only critical cases were admitted (such as CAD patients). However, for reporting purposes, the CAD incidence was 3,030.52 per 10,000 patients that year. According to a United Arab Emirates study, the incidence rate of major CAD per 10,000 person-years was 127, and higher systolic blood pressure (SBP) strongly predicted CAD in both men and women [12]. This agrees with the findings of our study, which revealed that the frequency rate of CAD among patients with high blood pressure was 66.3% per 1,364 patients in all age groups. The correlation between higher SBP and CAD in both men and women has been documented in previous studies [13,14].

CAD incidence rates vary globally among different high-risk populations. A recent five-year study in a neighboring Arab country found that the incidence rate of CAD among patients with diabetes was 17.6 per 10000 person-years, which is lower than the frequency rate determined in our study [12]. A study of Italian patients with diabetes found that men and women had higher CAD incidence rates of 288 and 233 per 10,000 person-years, respectively, than our study [15]. The prevalence rate of CAD among patients with diabetes was 61.3% in our study. In India, an 11-year population-based study of diabetes patients found a CAD incidence rate of 5.6 cases per 1000 person-years, which is lower than our study's frequency rate of CAD [16].

Previous studies reported that age is a major nonmodifiable risk factor linked to significant CAD occurrence [17,18]. In our study, age was a significant predictor of major CAD in men but not women, considering other risk variables [18,19]. In Europe and North America, the average age of patients with CAD is 60 to 65 years [3], higher than the average age of 56 years reported in a multicenter Middle Eastern population-based study [20]. The average age of the participants in our study was 59 years, indicating that the population at risk of CAD is younger than 60 years. This emphasizes the need for early detection of CAD and its risk factors.

Our findings revealed that younger patients with a smoking history (23.5% of the population) had a considerably greater risk of CAD than nonsmokers. Other classic cardiovascular risk variables (e.g., diabetes and serum lipids) are negatively influenced by cigarette smoking, and HTN has a multiplier effect on the incidence of CAD [21]. Smokers younger than 50 have a 10-fold increased risk of CAD compared to nonsmokers of the same age [22]. In our study, 19.4% of the participants were current smokers, and 16.3% were ex-smokers. In two previous studies, current smoking was significantly associated with increased risk, while ex-smokers had a higher risk than nonsmokers but lower than current smokers [23,24].

Risk factors analysis in this study found that 59% of patients under 60 have diabetes and 52.3% had a high HbA1c level. HbA1c reflects glucose control and is a significant predictor of CAD incidence in both sexes; CAD risk increases in patients as their HbA1c levels increase [23,24]. Diabetes is a significant risk factor for CAD [25].

In our study, 30.4% of the participants were obese. Being obese or overweight exacerbates or aggravates all atherogenic risk factors that predispose people to coronary events, regardless of age [26]. Current evidence suggests that BMI is independently related to CAD in patients with established coronary atherosclerosis and that the risk is enhanced even at mildly raised BMI levels [27].

The prevalence of high cholesterol and triglyceride levels in the present study were 20.2% and 15.5%, respectively. Previous research shows that a single risk factor (e.g., hypercholesterolemia or HTN) is responsible for the development of CAD [28]. Previous studies showed that a triglyceride level of 90 mg/dL raises the risk of CAD [22].

Studies in other countries showed positive connections between CAD and changes in population mean risk factors [23]. A longitudinal study may be required to demonstrate the effect of lifestyle changes by losing weight, increasing physical activity, quitting smoking, controlling HTN, controlling diabetes, and actively managing the metabolic syndrome in reducing the risk of CAD in Saudi Arabia [23,29].

Our study had several important limitations. First, this was a single-center study, and therefore a more extensive multicenter study is warranted to support our results. Secondly, restrictions due to COVID-19 precautions confounded our 2020 data. A repeat study where such pandemic-related restrictions are appropriately absent would strengthen the data.

## Conclusions

We conducted this study to assess the incidence of CAD from 2019 to 2020 in KAUH and to determine the major risk factors for CAD. We found a significant increase in the incidence of CAD. The increase was markedly observed among older male patients. Therefore, monitoring these variables in patients with CAD is advisable. Based on our results, a national community-based prevention effort should be implemented to avoid the expected CAD epidemic in Saudi Arabia. Measures to modify lifestyle and address metabolic syndrome management are required.
